# Low-Loss Pogo Pin Probe Card with a Coupling Isolation Structure up to 50 GHz

**DOI:** 10.3390/s23125420

**Published:** 2023-06-08

**Authors:** K. M. Lee, S. Ahn, E. Park, M. Kim

**Affiliations:** 1 School of Electrical Engineering, Korea University, Seoul 02841, Republic of Korea; leemin7@korea.ac.kr; 2 Samsung Electronics Co., Ltd., Suwon 16677, Republic of Korea; sanguck.ahn@samsung.com (S.A.);

**Keywords:** RF probe cards, mm wave RF testing, PCB circuit design, resonance removal technique

## Abstract

A design for a millimeter wave RF probe card that removes resonance is proposed. The designed probe card optimizes the position of the ground surface and the signal pogo pins to resolve the resonance and signal loss issues that occur when connecting a dielectric socket and a PCB. At millimeter wave frequencies, the height of the dielectric socket and pogo pin matches the length of half a wavelength, allowing the socket to act as a resonator. When the leakage signal from the PCB line is coupled to the 2.9 mm high socket with pogo pins, resonance at a frequency of 28 GHz is generated. The probe card uses the ground plane as a shielding structure to minimize this resonance and radiation loss. The importance of the signal pin location is verified via measurements in order to address the discontinuity caused by field polarity switching. A probe card fabricated using the proposed technique exhibits an insertion loss performance of −8 dB up to 50 GHz and eliminates resonance. A signal with an insertion loss of −3.1 dB can be transmitted to a system-on-chip in a practical chip test.

## 1. Introduction

As research on the 5G band continues, the importance of RF probes for the measurement of RF systems in millimeter wave on-wafer environments has grown. RF probes using tips fabricated via MEMS processes are commonly used in most on-wafer RF chip measurements [1]. In RF systems, such as RF integrated chips (ICs), a probe card structure that combines single probes is used for these measurements. However, the durability of commercial probe structures remains low, and they can be expensive. In addition, conventional probe cards that house bulky single probes are limited in terms of their shape and the number of array tips they can accommodate [2]. Advanced probe card pins fabricated using MEMS processing have been studied to solve this problem [3,4], but these are also characterized by certain limitations, such as the need for overdrive [5] during the measurement process and the susceptibility of the fine tips to being worn out [6]. As a result of these limitations, pogo pin products that use spring compression instead of RF probe tips have emerged as an alternative design choice that offers high durability. For instance, Yokowo produced a pogo pin probe card that can operate at frequencies of up to 6 GHz [7], and Pasternack sells a single pogo pin probe capable of measurements at up to 40 GHz [8]. However, the Yokowo pogo pin product is only suitable for low bands up to 6 GHz, and the wide 800 µm pitch of the Pasternack product makes it difficult to measure ICs on a wafer due to spatial limitations. Pogo pins are also advantageous due to their flexibility in arrangement. As a result, measurement systems utilizing pogo pins are suitable for RFIC tests that require multiple RF inputs and outputs. These characteristics can be applied to performance tests of MIMO communication systems developed to increase communication capacity over the millimeter wave band, as well as beam-forming system tests including tests of phased array antenna systems for beam steering. Consequently, the development of pogo pin systems operating at high frequencies is essential for the advancement of 5G bands.

Research on pogo pins that can be used in the RF band has recently received greater attention. In particular, there has been a focus on the use of pogo pins in the high-frequency band, based on the analysis of pogo pin characteristic impedance and the optimal pogo pin structure in various GHz bands [9] and the development of RF test models with pogo pins [10,11,12]. Although pogo pins are frequently used in low-frequency bands below MHz signals [13] without impedance analysis, analysis of the characteristic impedance of pogo pins themselves in the millimeter wave band is important. In recent studies, a method of directly connecting connectors to both ends of a pogo pin has been used to analyze the RF transmission characteristics of a pogo pin inserted into a dielectric socket. Using this method, the insertion loss performance up to the 10 GHz band was analyzed [14]. In another study, coupling loss of up to 10 GHz was analyzed by attaching connectors to each of two pogo pins [15]. Furthermore, to analyze pogo pin insertion loss at higher frequencies, a probe capable of transmitting and receiving higher-frequency signals than the connector was attached to both ends of a pogo pin to measure performance up to 20 GHz [16]. Studies have also been conducted on ground pin placement using simulations to reduce pogo pin coupling losses up to 10 GHz [17]. However, the limitation of previous studies is that they only analyzed the characteristics of the pogo pin itself.

In order to measure ICs using pogo pins, not only the pogo pin sockets but also the PCB line structure that transmits the external signal to the pogo pin are essential. Simply connecting the connector and the pogo pins, as in previous research, is very different from the actual pogo pin usage environment. To match the ball map of the IC, very closely spaced pin arrangements are required, and PCB lines are used to transmit signals to these pin arrangements. Therefore, in an actual testing environment, problems that were not observed in previous research may occur. In addition, because characteristic analysis has only been conducted for frequencies below 20 GHz, problems that may arise at high frequencies, such as in the millimeter wave band, have not been identified. The RF performance of pogo pins using pogo pin cables has also been investigated [18,19], demonstrating the possibility that pogo pins can operate up to millimeter wave bands in the 40 GHz band [20], but, in this case, the gap between the pogo pins was very wide at 25.4 mm, which is different from system-on-chip (SoC) tests.

In recent pogo pin research, pogo pins up to 30 GHz, including the 5G band, have been analyzed. In particular, research has analyzed the transmission and coupling performance of pogo pin arrays up to 30 GHz using only High-Frequency Structure Simulator (HFSS) simulation [21]. Another study developed a prototype pogo pin probe that can operate with an insertion loss of 20 dB up to 30 GHz [22]. However, the prototype pogo pin structure differed from actual test environments because the PCB used had a vertical structure. Research has also been conducted recently on probe cards using dielectric sockets combined with PCB lines [23], a structure that is similar to the probe cards used in practical testing. The problems that occur when a dielectric socket, pogo pins, and a PCB are combined were analyzed through measurements, with resonance issues observed at 28 GHz when the height of the dielectric socket matched half the wavelength and radiation issues occurring in the high-frequency band. The proposed solution involves using short pogo pins to raise the resonance frequency, but this has practical limitations in terms of the ease of fabrication and the loss of durability.

Based on the research to date, in the present paper, we aim to analyze the causes of resonance and high-frequency loss in a probe card with a fully assembled PCB, a dielectric socket, and 183 pogo pins. We subsequently propose a PCB design that utilizes an optimal ground plane and optimal ground pin location structure to overcome these resonance and high-frequency loss issues.

## 2. Probe Card PCB Design to Remove Resonance Issues

**Figure 1:**
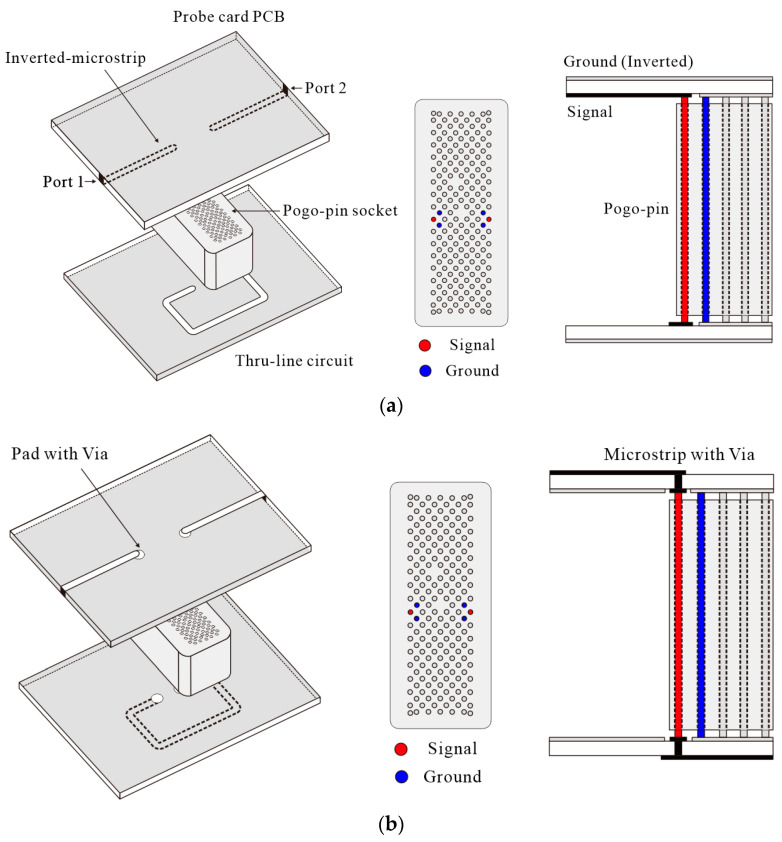

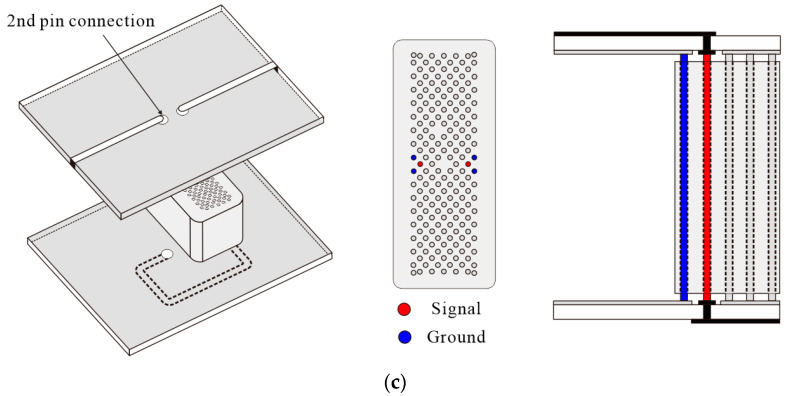
Three proof-of-concept models with vertical structure sketch for a probe card PCB simulated using HFSS: (**a**) inverted microstrip, (**b**) microstrip A, and (**c**) microstrip B configurations.

Two problems faced by prototype probe cards consisting of a dielectric socket and a PCB are the resonance that occurs when the socket height and pogo pin length are equal to half a wavelength at a specific frequency and radiation loss in the high-frequency band [23]. This resonance occurs because the dielectric resonator generates resonance by placing the dielectric material close to the PCB line, which allows the high-frequency signal leakage from the PCB line to couple with the dielectric structure. The leakage signal that enters the dielectric socket also creates resonance with the pogo pins inside the socket. Because the height of the pogo pins is the same as the socket height, parasitic resonance occurs around the resonator, resulting in resonance over a wider band. To verify this as the cause of the resonance, the dielectric resonator is modeled and simulated using HFSS (Figure 1). The simulation conditions are set to be the same as the environment connecting the probe card PCB line and the pogo pins. In particular, the HFSS model used in the simulation includes a dielectric socket fabricated from MDS100 that contains pogo pins and a Duroid 5880 substrate line.

**Figure 2:**
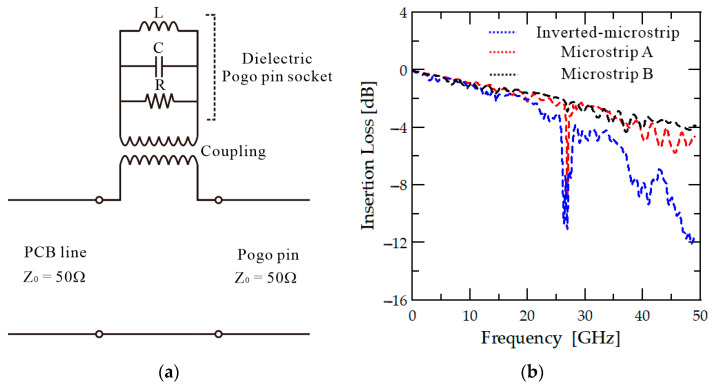
(**a**) Equivalent circuit model of dielectric pogo pin socket with PCB line, (**b**) HFSS simulation results for three PCB configurations.

The typical structure of the PCB is presented in Figure 1a. This structure is designed to allow direct contact between the PCB line and the signal pogo pin, which is colored red, to transmit RF signals, thus minimizing the reflection caused by the impedance mismatch between the two different types of line. The PCB line for the probe card uses an inverted microstrip structure in which the ground surface of the PCB is located on the top, and the line is located on the bottom near the dielectric socket with no isolation structure between the pogo pin socket and the line. This is not a problem in the low-frequency band because the leakage signal from the line is low, and the length of the pogo pins is relatively short. However, as the frequency increases, the conductor Q factor increases, and the overall Q factor, which determines the amount of radiation, also increases, resulting in higher radiation loss. All of the leakage from the bottom line is directed toward the dielectric socket, which leads to coupling loss. In order to evaluate this process, the structure presented in Figure 1a is simulated using HFSS, with the simulation results presented as the blue line in Figure 2b. Resonance occurs in the 28 GHz band when the length of the dielectric socket and pogo pins is equal to half of a wavelength, which results in an increase in loss.

The probe card structure, which consists of a dielectric socket and a PCB, can be represented by an equivalent circuit model, as shown in Figure 2a. In the high-frequency band, the dielectric socket acts as a dielectric resonator, whose resonance frequency is determined by its structure, size, and coupling with the PCB line [24]. The dielectric socket’s inductance, capacitance, and resistance components vary depending on its size, shape, and dielectric constant. Due to its complex structure, it is difficult to analyze the characteristics of the dielectric socket. It is also inefficient to connect matching sections to circuits to reduce resonance because the dielectric socket changes its shape flexibly depending on the test system. To solve the resonance problem in all cases, the most effective method is to remove the coupling between the dielectric pogo pin socket and the PCB line. The proposed probe card structure is designed to address this resonance issue by implementing a shield between the PCB line and the dielectric socket. This shielding prevents the coupling of signals in the dielectric material and is achieved by placing the ground surface of the PCB in the direction of the dielectric socket. Figure 1b shows that the ground surface is located at the bottom of the PCB to create a shielding structure, while the signal line is placed at the top. A via and pad are used to connect the pogo pins and the PCB line, and a small ground hole Is positioned around the pad. The ground plane Is designed to block radiation signals in the downward direction. Using this structure, signal coupling between the dielectric socket and the PCB line can be reduced. The structure presented in Figure 1b is simulated using HFSS to verify the proposed method, and the results are displayed as the red line in Figure 2b, showing that only the high Q factor resonance in the 28 GHz frequency range remains. In the simulation of this microstrip line structure, the via structure added to connect the pogo pins and the PCB line results in a slight increase in the insertion loss due to the increased mismatch.

If the ground surface is placed at the bottom of the PCB, the coupling between the PCB line and the dielectric socket is weakened. However, when using the proposed microstrip design denoted as microstrip A in this paper in a traditional manner, with the outermost pogo pin used as the signal pin, other problems may occur. In general, the pogo pin located in the first column of the array is primarily used as the RF signal pin (the red pins in Figure 1a). This is because the outermost pin can be connected to the PCB line in the most simple and direct manner. Using internal pins can be challenging because the previous probe card PCB was designed with a typical inverted microstrip line structure in which the PCB line and pin are directly connected. If this connection method is applied to microstrip A, the signal line is connected to the pogo pin in the first column, and the ground surface is connected to the pin in the second column. In this case, the signal line and the ground surface are reversed. Therefore, at the connection point between the PCB line and the pogo pin, the E-field polarity of the transmission line is also reversed. As a result, the field polarity switches at both ends of the pogo pin, causing discontinuities at two points and leading to resonance caused by the length of the pogo pin. To eliminate the additional resonance that occurs in this PCB structure, an optimal position for the signal pogo pin is proposed.

Connecting the second column pin to the signal line and the first column pins to the ground plane, as shown in Figure 1c, eliminates the field polarity reversal of the E-field. Therefore, if the pin in the second column is used as the signal pin (the red pin in Figure 1c), the resonance can be removed. An HFSS simulation is conducted to test this hypothesis, and the results are presented as the black line in Figure 2b. As shown by the red line, when the field polarity is switched, high Q resonance occurs at 28 GHz. However, as indicated by the black line, when this field polarity switching is avoided, resonance does not occur.

## 3. Fabrication of a Prototype Probe Card for Verification

**Figure 3:**
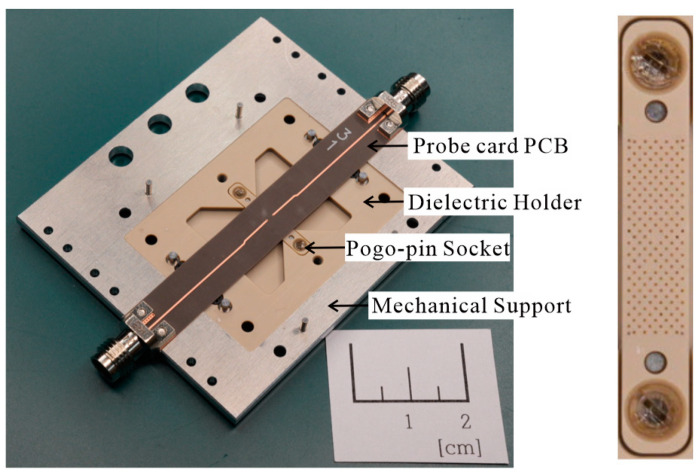
Photographs of the assembled prototype probe card and pogo pin socket.

A prototype RF probe card based on the proposed design is fabricated for the automatic testing of an RF SoC in the millimeter wave frequency band up to 50 GHz. It consists of a dielectric socket containing 183 pogo pins, a probe card PCB for transmitting signals to the pogo pins, a dielectric holder for fixing the socket, and a metal holder for mechanical support and packaging. The fabricated prototype probe card is depicted in Figure 3. The dielectric socket utilized in the study contains an array of 183 pogo pins located in the center of the probe card, with a pin-to-pin spacing of 0.35 mm. The spacing of the pogo pins is designed to ensure that the characteristic impedance of the transmission line, consisting of a single signal pogo pin and two peripheral ground pins, is 50 Ω.

The pogo pins used in this study are the low-loss GN855AMR-DGPC model produced by Leeno Industrial Inc. The dielectric socket is made from low-loss MDS100 material to minimize the material loss. Of the 183 pins, two pairs of pins are used as signal pins (Figure 1). The pin in the first column is typically used as an RF signal pin in a conventional probe card, while the pin in the second column is used for signal transmission in the proposed probe card. The PCB used in the probe card consists of a 5 mil low-loss Duroid 5880 substrate to minimize material and high-frequency radiation loss. The dielectric holder is also made from the same MDS100 material as the dielectric socket to further reduce the material loss. The dielectric holder is used to secure the socket in place and isolate the measurement environment around it. The metal holder is designed to secure the probe card PCB, dielectric holder, and RF connector in a single prototype probe card using guide pins and screws. All components of the probe card are assembled tightly to minimize the measurement loss caused by contact issues or deformation of the thin PCB substrate. To prevent any negative impact on the RF line in the PCB, the metal holder is designed to remain over 1 λ from the line, as confirmed through HFSS simulations. The SMA connector used in the probe card is an OS-50 connector that can be used for measurements up to 50 GHz. A Hirose H24LRSR2 product is used in the final probe card, which offers the lowest loss characteristics for measurements up to 50 GHz. This connector can be fixed to the substrate and metal holder using screws, minimizing contact loss.

## 4. Probe Card Measurements

**Figure 4:**
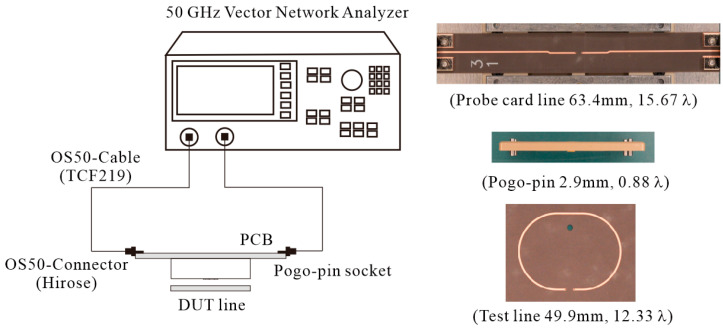
Probe card measurement setup and photograph of the prototype probe card and test line.

In this study, a prototype pogo pin probe card is fabricated and tested to validate the proposed resonance removal strategy. The measurement setup is illustrated in Figure 4, and the S-parameters are measured using a 50 GHz vector network analyzer (VNA; Keysight E8364B). The signal transmission performance of the pogo pin probe card is analyzed by measuring the insertion loss for a two-port system, including the through-test line. The total length of the structure, including the through-test line, is 29 λ at 50 GHz (Figure 4). The entire measurement system is composed of a dielectric socket containing pogo pins, a probe card PCB, and a 50 Ω test line for the through test. Both the probe card PCB and test line are fabricated using a 5 mil Duroid 5880 substrate.

**Figure 5:**
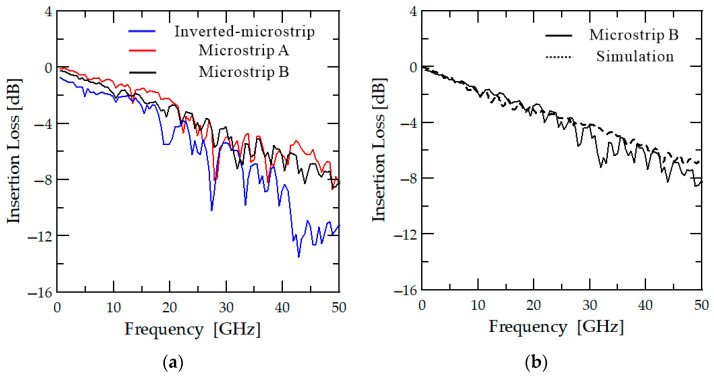
(**a**) Insertion loss measurement results for three probe cards, and (**b**) the measurement (solid line) and simulation (dashed line) results for the proposed probe card.

The results obtained after applying the techniques described in Section 2 are presented as the black line in Figure 5. It can be observed that the transmission loss follows a linear trend without any resonance at 28 GHz or high-frequency losses, which were problems in previous research [23]. The insertion loss for the probe card is measured to be 8.1 dB at 50 GHz. A simulation conducted under the same conditions as the measurements (the black dashed line in Figure 5b) produces a similar trend to the measured results. However, during the actual measurements, a higher ripple loss is observed when compared with the simulation results. This ripple loss leads to a 1.8 dB difference between the simulation and measurement results at 40 GHz. The loss is thought to be caused by the contact between the pogo pins and the PCB line, which is estimated to be 0.45 dB per contact. Although the loss is higher than the 2.7 dB loss observed for the commercial RF probe, it is believed that the actual loss difference can be reduced further if the line is shortened. The reflection loss for the probe card is below 6.7 dB across the entire band. The reflection performance is limited by the manufacturing of vias and pad structures connecting the PCB line and pogo pins. Thinner vias and pads are needed to achieve a 50 Ω impedance match, but the diameter of the vias is limited by the PCB manufacturing process to a minimum of 0.2 mm, and the pads have a manufacturing limit of 0.4 mm. As a result, perfect matching is not possible. However, we believe that this problem can be easily resolved by improving the manufacturing precision.

To analyze the substrate loss for the probe card PCB line and the through-test line, which are the main contributors in the overall measurement system of the probe card, a straight transmission line of 65 mm in length is fabricated. The length is the same as that of the probe card PCB. The line is designed to have the same microstrip structure as the probe card, with a width of 0.365 mm, to produce a characteristic impedance of 50 Ω. The S-parameters are measured using a 50 GHz VNA, and the insertion loss is found to be 1.9 dB at 40 GHz. No resonance is observed in the simple line, and the reflection loss is measured to be below −16 dB across the entire frequency band. A loss tangent value of 0.006 is obtained from fitting the 40 GHz data. The measured line loss data can be used to estimate the proportion of the signal that enters the probe card and reaches the chip.

The proportion of the signal that reaches the chip can be calculated by taking half of the value from the measured probe card insertion loss, excluding the loss of the through-test line. This value can be considered the insertion loss that reaches the SoC that is to be measured through the probe card, including the probe card PCB line and the pogo pins, when the external signal is input into the probe card. At a frequency of 50 GHz, the signal reaching the chip is calculated to be −3.1 dB. This means that half of the RF input power to the probe card is transmitted to the chip. Table 1 presents the comparison of the performance of multiple-pin probes. The probe card proposed in this paper achieved a low-loss performance up to 50 GHz band, which is higher than the performance of existing pogo pin.

To determine whether the transmission performance of the probe card has improved, typical inverted microstrip and microstrip A probe card PCBs are also produced and tested. The results of the measurements are presented in Figure 5. In Figure 5a, the blue solid line represents the results obtained using the typical inverted microstrip line PCB with the ground at the top. Similar to in previous research [23], strong resonance occurs at a frequency of 28 GHz, and there is high-frequency band loss over 40 GHz. On the other hand, the PCB structure in which the ground surface is located at the bottom with a basic microstrip line exhibits reduced loss in the high-frequency band, and the resonance is weakened (the red line in Figure 5a). It is confirmed that the coupling between the PCB line and the dielectric socket causes a dielectric resonator problem. The red line in Figure 5a shows that weak resonance remains at a frequency of 28 GHz, but this resonance disappears when the polarity switch of the field is removed by optimizing the signal pin position, as shown by the black line in Figure 5a.

The proposed probe card PCB aims to reduce radiation loss in the high-frequency band by using the thinnest possible substrate. Therefore, for the final probe card, a 5 mil thick Duroid substrate is used. In previous research [23], a 10 mil thick substrate was used, and resonance-related losses were higher in the 28 GHz band. In contrast, the 5 mil thick substrate used in the present study results in reduced loss compared to this previous study.

**Figure 6:**
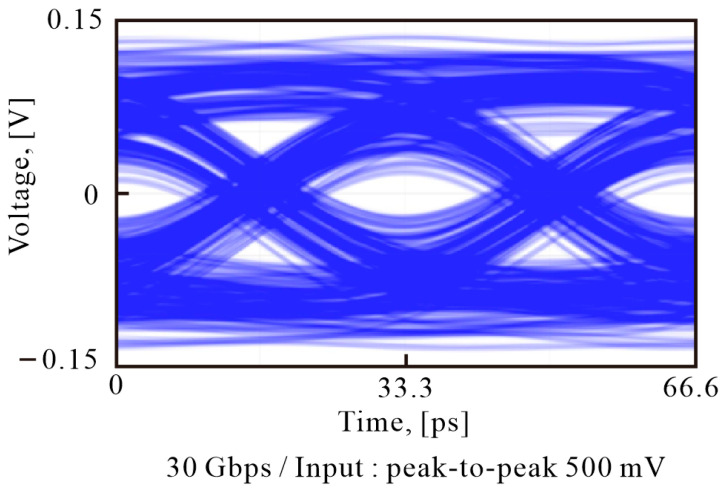
Measured eye diagram for the 30 Gbps OOK modulation of the proposed probe card.

We use a 32 GHz arbitrary waveform generator (AWG, Keysight M8195A) and a 33 GHz oscilloscope (Keysight UXR0334A) to measure the 30 Gbps OOK modulation signal of the RF probe card. The signal transmission performance of the proposed probe card is measured by delivering a peak-to-peak voltage of 0.5 V to the probe card, and the measurement results are presented in Figure 6. The probe card is able to deliver signals of tens of Gbps in the 5G band without any problems, indicating that it can be used to measure the performance of 5G band communication chips in the future.

## 5. Conclusions

A prototype probe card that utilizes resonance-reducing techniques is fabricated and tested. The probe card includes a dielectric pogo pin socket and a PCB that transmits signals to the pogo pins. The goal of the analysis is to present solutions to the resonance problem that arises in the millimeter wave frequency band when these components are combined. The presence of signal coupling, which causes the dielectric resonance problem and the loss in the millimeter wave frequency band, is confirmed in the measurements. The problem is resolved through the design of the PCB rather than the dielectric socket, which is difficult to modify. The ground surface of the PCB is utilized as a radiation signal shielding structure to minimize coupling, and an optimal signal pin arrangement is proposed to address the issue. A probe card PCB is then fabricated with two structures, and hypothesis verification is conducted using the measurements. As a result, a prototype probe card is developed that can transmit the input signal to the SoC chip with an insertion loss of only −3.1 dB, and a target insertion loss of −2.3 dB can be achieved at a frequency of 28 GHz. However, due to the 65 mm length of the probe card, only about 50% of the signal can be transmitted to the chip. To further improve this transmission performance, methods such as miniaturizing the length of the dielectric socket can be implemented. By analyzing the cause of problems occurring at high-frequency bands and proposing a simple technique to solve them, we aim to provide practical testing facilities that can be quickly implemented. These techniques can be easily applied to industries that use frequencies above 5G, making them accessible and widely applicable.

## Figures and Tables

**Table 1:** Performances of multiple-pin RF probes.

Ref.	Max. Frequency	No. Tips	Type	Pitch	Insertion Loss
[2]	40 GHz	100	MEMS Needle	100 mm	1 dB @38.6 GHz
[7]	6 GHz	250	Pogo pin	130 mm	-
[8]	40 GHz	3	Pogo pin	800 mm	0.5 dB @40 GHz
[14]	10 GHz	12	Pogo pin	700 mm	-
[16]	20 GHz	36	Pogo pin	800 mm	3 dB @20 GHz
[18]	6 GHz	101	Pogo pin	1270 mm	2 dB @5 GHz
[20]	35 GHz	16	Pogo pin	2540 mm	9 dB @35 GHz
[22]	20 GHz	3	Pogo pin	464 mm	12 dB @20 GHz
[23]	25 GHz	157	Pogo pin	350 mm	0.5 dB @25 GHz
This work	50 GHz	183	Pogo pin	350 mm	3.1 dB @50 GHz
